# Parasites and chronic renal failure

**DOI:** 10.12861/jrip.2014.25

**Published:** 2014-12-01

**Authors:** Reza Mohammadi Manesh, Ahmad Hosseini Safa, Seyedeh Maryam Sharafi, Rasool Jafari, Mehran Bahadoran, Morteza Yousefi, Hamid Nasri, Hossein Yousofi Darani

**Affiliations:** ^1^Department of Parasitology and Mycology, Isfahan University of Medical Sciences, Isfahan, Iran; ^2^Infectious Diseases and Tropical Medicine Research Center, Isfahan University of Medical Sciences, Isfahan, Iran; ^3^Student Research Committee, Shahrekord University of Medical Sciences, Shahrekord, Iran; ^4^Department of Internal Medicine, Isfahan University of Medical Sciences, Isfahan, Iran; ^5^Infectious Diseases and Tropical Medicine Research Center, Isfahan University of Medical Sciences, Isfahan, Iran

**Keywords:** Parasitic infections, Renal disorders, Renal transplantation

## Abstract

Suppression of the human immune system results in an increase in susceptibility to infection by various infectious agents. Conditions such as AIDS, organ transplantation and chronic renal insufficiency (CRI) are the most important cause of insufficient immune response against infections. Long term renal disorders result in uremia, which can suppress human immune system. Parasitic infections are one of the most important factors indicating the public health problems of the societies. These infections can be more hostile and life threatening in susceptible individuals than in the normal people. In these patients some parasitic infections such as blastocystiosis, cryptosporidiosis and toxoplasmosis have been reported to be more prevalent. This review aimed to give an overview about parasitic infections in patients with renal disorders.

**Implication for health policy/practice/research/medical education:**

Chronic renal failure is one of the causes of immune suppression. In these patients some parasitic infections such as blastocystiosis, cryptosporidiosis and toxoplasmosis have been reported to be more prevalent. This review aimed to give an overview about parasitic infections in patients with renal disorders.

## Introduction

By increasing the life expectancy among mankind population, subsequent incidence of some chronic diseases, such as chronic renal insufficiency (CRI) is increasing (1). CRI itself results some complications, in which renal function disturbs. When the renal function lost, body cannot sustain equilibrium of electrolytes. Consequently uremia may occur (2), which is the result of excess non-excreted metabolites accumulation, like urea (3). Natural state of immune-depression can cause by uremia and subsequent increase in the incidence of infections may happen (4,5). Around 48% of deaths in CRI patients are associated with these infections (2,6).

Also reduced immune response to various antigens, has been detected in patients with chronic kidney disease (CKD) who undergoing dialysis treatment (7-15). Additionally, it has been found that sepsis-related death is between 100 and 300-times more frequent in patients undergoing hemodialysis comparing to the general population (16).

Intestinal parasitic infections have been considered as one of the major public health problems, predominantly in developing countries. However, because of some immunological conditions such as intentional suppression of immune response and also some immunodeficiencies in developed countries, some of these parasites have become a problematic issue (17). Chronic renal diseases are also one of the causes of insufficient immune response to the infections (2,18).

Considering the important role of parasitic infections in creating complications or deaths in patients with renal problems, in this paper parasitic infections in patients with chronic renal disease is reviewed.

## Intestinal parasites

There are lots of studies indicating higher prevalence of intestinal parasitic infections in patients with renal disorders, which some are reviewed.

From 2006 to 2007 hemodialysis patients of Campo Mourão city, Brazil were examined for *Blastocystis* sp. and other intestinal parasites and also the associated diarrhea. Thirty three (45.1%) out of 86 hemodialysis patients and 36 (25.7%) out of 146 control individuals were found to be infected by the parasites. The prevalence of intestinal parasitic infections in hemodialysis patients has been reported significantly higher than the healthy individuals. It is suggested that parasitological stool examinations, particularly for *Cryptosporidium* spp. and *Blastocystis* sp., are better to be perform in routine medical follow up examinations of hemodialysis patients (19).

In another study the prevalence of amoebiasis caused by *Entamoeba histolytica/dispar* in chronic hemodialysis patients has been reported to be about 8% (20). In Turkey a study carried out on patients with an end-stage renal failure and 150 healthy volunteers. Parasites were found in 62 (43.7%) out of 142 dialysis patients and 19 (12.7%) out of 150 healthy controls. The most prevalent cause of parasitic infection in the dialysis patients were *Blastocystis* sp. (23.9%), *Giardia lamblia* (8.5%), *E. histolytica* (2.1%), *Microsporidia* spp. (2.1%), and *Cryptosporidium* spp. (2.1%) (21). Intestinal parasitic infections were more prevalent in clinically asymptomatic renal transplant recipients than in patients with chronic haemodialysis and healthy individuals (22).

Study of Gill *et al.,* in 2013, illustrated that 51.6% of hemodialysis patients were infected by intestinal parasites, but the prevalence of the infections in control group was 61.6%. *Cryptosporidium* antigen was observed only in patients with CKD (24.5%), but not in controls (23). They reported prevalence of 24.5% for cryptosporidiosis, which was considered to be high, even higher than some of the reports from renal transplant recipient patients (24) and livestock owners (25,26), which were at risk of infection. Their study indicated that patients with CKD were at risk of infection with *Cryptosporidium* spp. (23).

The prevalence of *Cryptosporidium* infection in patients with end-stage of chronic renal failure was investigated and compared to the healthy controls. Fifteen out of 174 patients with dialysis was positive for *Cryptosporidium*, while none of the controls had the infection. The reported prevalence was significantly higher in dialysis patients (27).

In another study prevalence of *Cryptosporidium* infection among renal transplant recipients, patients referred to hemodialysis and the control group reported 34.8%, 25% and 17.4%, respectively (28). In the study carried out in Hamadan city, Iran, the prevalence of cryptosporidiosis has been reported 0.55% in renal transplanted patients, which is controversial to the previously mentioned studies (24).

Also a very interesting report indicated that the prevalence of *Cryptosporidium* infection was 80% in patients with cancer, 25% in diabetics and 35% in dialysis patients (29), which are considerably higher than those in the reports from general population and diarrheic children (25,30). In other studies 11.5% of renal transplant and 3.88% of hemodialysis patients were positive for *Cryptosporidium* infection. No positive results were found in the control groups (31).

Considering spore-forming protozoa, including: *Isospora belli*, *Cryptosporidium parvum*, *Microsporidia* and *Cyclospora cayetanensis* in patients with CKD, it has been reported that 33.3% of these patients were infected by these parasites (32). In another study, fecal samples of 104 chronic hemodialysis patients and 140 healthy controls were investigated for cryptosporidiosis. The prevalence of *Cryptosporidium* infection observed higher (11.5%) in dialysis patients compared to controls (3.6%) (33).

## Tissue parasites

Anti-*Toxoplasma* IgG has been reported in 27.3% and 3.6% of 205 dialysis patients with renal insufficiency and 360 healthy controls, respectively (34). In another similar study, statistically significant higher prevalence has been observed for anti-*Toxoplasma* IgG and IgM seropositivity in hemodialysis patients than controls. So it has been recommended that hemodialysis patients should be tested for anti-*Toxoplasma* IgG and IgM using specific serological tests in order to prevent the disseminated infection (35).

Other report showed that 36.8% of renal failure patients and 10.5% of normal population were seropositive for anti-*Toxoplasma* IgG and IgM. Also 56.7% and 16.7% of patients with regular hemodialysis and 69% and 24.1% of renal transplanted patients were seropositive for anti-*Toxoplasma* IgG and IgM, respectively (36). Higher rates of anti-*Toxoplasma* IgG and IgM, in renal transplanted and renal failure patients have also been reported (37,38). In another investigation significant higher sero-prevalence of toxoplasmosis has been reported in patients with chronic renal failure (38.3%) compared to the healthy controls (15%) (39).

In a study carried-out in Ahvaz city, southwest of Iran, 29.3% and 7.9% of hemodialysis patients and 26% and 4% of controls were seropositive for anti-*Toxoplasma* IgG and IgM, respectively (40). Also in other work the prevalence of anti-*Toxoplasma gondii * antibodies in 173 hemodialysis patients with CKD and 40 healthy controls were 56.06% and 1.73% for hemodialysis patients and 20% and 0% for healthy controls, respectively (41). Finally anti-*Leishmania donovani* antibodies among 1500 multiply transfused hemodialysis patients was (37%) and among 1194 volunteer blood donors was (9%) (42).

## Exoparasites

*Demodex folliculorum* infestation has been detected in 25.53% of CKD patients and 18.42% of healthy controls (43). Also the *D. folliculorum * infection reported in 19.54% of dialysis patients and in 10.34% of healthy controls (44). The mean mite count in patients with *infestation* who were in end stage of renal failure was 6.12/cm^2^ while in normal controls it was 0.31/cm^2^ (45).

## Case reports about parasites in patients with renal disorders

Case report studies about parasites in patients with renal disorders are summarized in  Table 1.

**Table 1:** Parasites reported in hemodialysis patients in case report studies

Parasite reported	References
Strongyloides stercoralis	(46-48)
Leishmania spp	(49,50)

## Conclusion

In conclusion, some parasitic diseases are considered as public health problems especially in developing countries (37,38,51,52). Patients with chronic kidney diseases are susceptible to be infected by some parasites such as *Blastocystis* sp., *Cryptosporidium* spp., and *Toxoplasma gondii*. Parasitic infections in these patients may make their situation much more complicated. Continues parasitological monitoring of patients with chronic renal failure is recommended for the possible control of the infections.

## Authors’ contributions

All authors contributed the manuscript equally.

## Ethical considerations

Ethical issues (including plagiarism, informed consent, misconduct, double publication and redundancy) have been completely observed by authors.

## Conflict of interests

The authors declared no competing interests.

## Funding/Support

None.

## References

[1] Lessa I, Mendonca GA, Teixeira MTB (1996). [Noncommunicable chronic diseases in Brazil: from risk factors to social impact]. Boletín de la Oficina Sanitaria Panamericana (OSP).

[2] Marques AB, Pereira DC, Ribeiro R (2005). [Reasons and frequency of CRF patients’ hospitalization in hemodialysis treatment]. Arq Ciênc Saúde.

[3] Levin A, Rocco M (2007). KDOQI clinical practice guidelines and clinical practice recommendations for diabetes and chronic kidney disease. Am J Kidney Dis.

[4] Kauffman CA, Manzler A, Phair J (1975). Cell-mediated immunity in patients on long-term haemodialysis. Clin Exp Immunol.

[5] D’avila R, Guerra EMM, Rodrigues CIS, Fernandes FA, Cadaval RAM, Almeida FA (1999). Sobrevida de pacientes renais crônicos em diálise peritoneal e hemodiálise. J Bras Nefrol.

[6] Barbosa DA, Gunji CK, Bittencourt AR, Belasco AG, Diccini S, Vattimo F (2006). [Co-morbity and mortality of patients in dialysis treatment]. Acta Paul Enferm.

[7] Eleftheriadis T, Antoniadi G, Liakopoulos V, Kartsios C, Stefanidis I. Basic science and dialysis: disturbances of acquired immunity in hemodialysis patients. Paper presented at: Seminars in dialysis 2007. 10.1111/j.1525-139X.2007.00283.x 17897251

[8] Vanholder R, DeSmet R, Hsu C, Vogeleere P, Ringoir S. Uremic Toxins-the middle molecule hypothesis revisited. Paper presented at: Seminars in nephrology 1994. 8036355

[9] Newberry WM, Sanford JP (1971). Defective cellular immunity in renal failure: depression of reactivity of lymphocytes to phytohemagglutinin by renal failure serum. J Clin Invest.

[10] Schindler R, Linnenweber S, Schulze M, Oppermann M, Dinarello CA, Shaldon S (1993). Gene expression of interleukin-1β during hemodialysis. Kidney Int.

[11] Girndt M, Heisel O, Köhler H (1999). Influence of dialysis with polyamide vs haemophan haemodialysers on monokines and complement activation during a 4-month long-term study. Nephrol Dial Transplant.

[12] Kaneko T, Osono E, Hayama N, Lino Y, Terashi A (1997). T-cell activation modified by parathyroid hormone (PTH) in patients with end-stage renal disease. Clin Nephrol.

[13] Yang S, Smith C, Prahl JM, Luo X, Deluca HF (1993). Vitamin D Deficiency Suppresses Cell-Mediated Immunity in Vivo. Arch Biochem Biophys.

[14] Giacchino F, Belardi P, Coppo R (1981). Effects of blood transfusion on cellular immunity. Proc Eur Dial Transplant Assoc.

[15] Hoen B, Kessler M, Hestin D, Mayeux D (1995). Risk factors for bacterial infections in chronic haemodialysis adult patients: a multicentre prospective survey. Nephrol Dial Transplant.

[16] Sarnak MJ, Jaber BL (2000). Mortality caused by sepsis in patients with end-stage renal disease compared with the general population. Kidney Int.

[17] Ferreira MS (2000). Infections by protozoa in immunocompromised hosts. Memórias do Instituto Oswaldo Cruz.

[18] Eknoyan G, Levin NW (2002). K/DOQI clinical practice guidelines for chronic kidney disease: evaluation, classification, and stratification. Am J Kidney Dis.

[19] Kulik RA, Falavigna DL, Nishi L, Araujo SM (2008). Blastocystis spand other intestinal parasites in hemodialysis patients. Braz J Infect Dis.

[20] Ferreira-Filho SR, da Costa Braga FC, de Sa DM, Nunes EB, Parreira Soares JS, Padovese SM (2011). Entamoeba histolytica/Entamoeba dispar infection in chronic hemodialysis patients. Saudi J Kidney Dis Transpl.

[21] Karadag G, Tamer GS, Dervisoglu E (2013). Investigation of intestinal parasites in dialysis patients. Saudi Med J.

[22] Esposito R, Orlando G, Inzoli C, Calello G, Moroni M, Rivolta E (1986). Asymptomatic parasitic infections in renal transplant recipients and in haemodialysis patients. Boll Ist Sieroter Milan.

[23] Gil FF, Barros MJ, Macedo NA, Júnior CG, Redoan R, Busatti H (2013). Prevalence of Intestinal Parasitism and Associated Symptomatology among Hemodialysis Patients. Rev Inst Med Trop Sao Paulo.

[24] Jafari R, Gharibi Z, Fallah M (2014). The prevalence of cryptosporidium infection among renal transplanted patients in Hamadan city, West of Iran. Avicenna J Clin Microb Infec.

[25] Jafari R, Maghsood AH, Fallah M (2012). Prevalence of Cryptosporidium Infection among Livestock and Humans in Contact with Livestock in Hamadan District, Iran, 2012. J Res Health Sci.

[26] Jafari R, Maghsood AH, Safari M, Latifi M, Fallah M. Comparison of fecal antigen detection using enzyme linked immunosorbent assay (ELISA) with auramine-phenol staining method for diagnosis of human cryptosporidiosis. Jundishapur J Microbiol. In press. 10.5812/jjm.16470 PMC4376972 25825642

[27] Turkcapar N, Kutlay S, Nergizoglu G, Atli T, Duman N (2002). Prevalence of Cryptosporidium infection in hemodialysis patients. Nephron.

[28] Chieffi PP, Sens YA, Paschoalotti MA, Miorin LA, Silva HGC, Jabur P (1998). Infection by Cryptosporidium parvum in renal patients submitted to renal transplant or hemodialysis. Rev Soc Bras Med Trop.

[29] Baqai R, Anwar S, Kazmi S (2005). Detection of cryptosporidium in immunosuppressed patients. J Ayub Med Coll Abbottabad.

[30] Fallah M, Haghighi A (1996). Cryptosporidiosis in children with diarrhea submitted to health centers in the west of iran (hamedan). Med J Islam Repub Iran.

[31] Tappeh KH, Gharavi M, Makhdoumi K, Rahbar M, Taghizadeh A (2006). Prevalence of Cryptosporidium sppInfection in Renal Transplant and Hemodialysis Patients. Iran J Public Health.

[32] Ali M, Mahmoud L, Abaza B, Ramadan M (2000). Intestinal spore-forming protozoa among patients suffering from chronic renal failure. J Egypt Soc Parasitol.

[33] Seyrafian S, Pestehchian N, Kerdegari M, Yousefi Ha, Bastani B (2006). Prevalence rate of Cryptosporidium infection in hemodialysis patients in Iran. Hemodial Int.

[34] Tong D, Yang J, Xu G, Shen G (2011). [Serological investigation on Toxoplasma gondii infection in dialysis patients with renal insufficiency]. Zhongguo xue xi chong bing fang zhi za zhi.

[35] Ocak S, Duran N, Eskiocak AF, Aytac H (2005). Anti-Toxoplasma gondii antibodies in hemodialysis patients receiving long-term hemodialysis therapy in Turkey. Saudi Med J.

[36] Aufy S, Mahgoub A, Saadi M, Adel EM (2009). Serological detection of Toxoplasma gondii in chronic renal failure patients and renal transplant recipients. J Egypt Soc Parasitol.

[37] Jafari R, Sadaghian M, Safari M (2012). Seroprevalence of Toxoplasma gondii Infection and Related Risk Factors in Tabriz City, Iran, 2008. J Res Health Sci.

[38] Rasouli S, Sadaghian M, Jafari R (2014). Prevalence of human toxoplasmosis and related risk factors using Electrochemiluminescence (ECLIA) method in West Azarbaijan Province, Iran, 2010. Int J Biosci.

[39] Abbas M, Zaki M, Afify N (1996). Prevalence of Toxoplasma gondii and cytomegalovirus antibodies in patients with chronic renal failure. J Egypt Soc Parasitol.

[40] Saki J, Khademvatan S, Soltani S, Shahbazian H (2013). Detection of toxoplasmosis in patients with end-stage renal disease by enzyme-linked immunosorbent assay and polymerase chain reaction methods. Parasitol Res.

[41] Demirtaş F, Yalçin Ş, Yaman O, Tokgöz B, Utaş C, Şahin İ (2003). Anti-Toxoplasma gondii antibodies in haemodialysis patients with chronic renal failure. Yonsei Med J.

[42] Luz KG, da Silva VO, Gomes EM, Machado FC, Araujo MA, Fonseca HE (1997). Prevalence of anti-Leishmania donovani antibody among Brazilian blood donors and multiply transfused hemodialysis patients. Am J Trop Med Hyg.

[43] Özçelik S, Sümer Z, Değerli S (2007). [The Incidence of Demodex folliculorum in Patients with Chronic Kidney Deficiency]. Türkiye Parazitol Derg.

[44] Yagdiran Düzgün O, Aytekin S (2007). Comparison of Demodex folliculorum density in haemodialysis patients with a control group. J Eur Acad Dermatol Venereol.

[45] Karincaoglu Y, Esrefoglu Seyhan M, Bayram N, Aycan O, Taskapan H (2005). Incidence of Demodex folliculorum in patients with end stage chronic renal failure. Ren Fail.

[46] Kosmadakis G, Georgoulias C, Filiopoulos V, Stefanou I, Smirloglou D, Michail S (2010). Lethal Strongyloides stercoralis superinfection in an immunocompromised patient. Ren Fail.

[47] Said S, Nevez G, Moriniere P, Fournier A, Raccurt C (1998). [Hemodialysis and strongyloidiasis: a presumed cause of eosinophilia able to mask the other]. Nephrologie.

[48] Hachim K, Mortajil F, Chakib A, Ramdani B, Zaid D (2005). [Severe anguilluliasis in a chronic haemodialysis patient]. Presse Med.

[49] Hussein MM, Mooij JM, Roujouleh HM, Hamour AO, Felemban H (2001). Non-typhoid Salmonella septicemia and visceral leishmaniasis in a renal transplant patient. Transplantation.

[50] Maggi P, Larocca AM, Mininni F, Fiorentino G, Saracino AL, Chironna M (2002). Autochthonous mucosal leishmaniasis in a hemodialyzed Italian patient. New Microbiol.

[51] Imani R, Khoshde A, Darani HY, Mobasheri M (2013). Comparative Clinical Trial of Mebendazole, Praziquantel and Metronidazole in Treatment of Human Giardiasis. International Journal of Travel Medicine & Global Health.

[52] Yousofi Darani H (2008). Situation of hydatid cyst infection during last two decades (1985-2005) in Iran. J Shahrekord Univ Med Sci.

